# The emerging role of microRNAs in Alzheimer's disease

**DOI:** 10.3389/fphys.2015.00040

**Published:** 2015-02-12

**Authors:** Grazia D. Femminella, Nicola Ferrara, Giuseppe Rengo

**Affiliations:** ^1^Department of Translational Medical Sciences, Federico II UniversityNaples, Italy; ^2^“Salvatore Maugeri” Foundation - Istituto di Ricovero e Cura a Carattere Scientifico - Scientific Institute of Telese TermeTelese Terme, Italy

**Keywords:** microRNAs, Alzheimer's disease, biomarker, neurodegeneration, functional recovery, gene expression

## Abstract

MicroRNAs (miRNAs) are small non-coding RNA which have been shown to regulate gene expression. The alteration ofmiRNAs expression has been associated with several pathological processes, including neurodegeneration. In the search for easily accessible and non-invasive biomarkers for Alzheimer's disease (AD) diagnosis and prognosis, circulating miRNAs are among the most promising candidates. Some of them have been consistently identified as AD-specific miRNAs and their targets also seem implicated in pathophysiological processes underlying AD. Here, we review the emerging role for miRNA in AD, giving an overview on general miRNAs biology, their implications in AD pathophysiology and their potential role as future biomarkers.

Alzheimer's disease (AD) is a progressive neurodegenerative disorder and the most common form of dementia in the world. AD affects more than 20% of individuals over 80 years of age, and epidemiological data predict that by 2050 over 35 millions of people will be affected, with a significant social and economic burden (Danborg et al., 2014). Clinically, AD is typically characterized by progressive memory loss, impairment of other cognitive functions and inability to perform activities of daily living. The earliest clinical stage of AD is defined as mild cognitive impairment (MCI), and it is characterized by impairment in memory and/or others cognitive domains but preserved functional abilities, with an annual conversion rate from MCI to AD of 15%. To date, the available therapeutic agents are only able to slow disease progression, with limited benefits (Kim et al., 2014). Thus, there is an urgent need to identify new potential biomarkers which could help in early diagnosis at the prodromal stages.

Pathologically, AD brain is characterized by two major protein abnormalities: extracellular amyloid-β (Aβ) deposition and intracellular neurofibrillary tangles (NFT) formation, both ultimately leading to extensive neuronal degeneration. Aβ peptides derive from sequential cleavage of membrane-spanning amyloid precursor protein (APP) by beta-site APP cleaving enzyme 1 (BACE1) and the γ-secretase complex containing the presenilin (PSEN) proteins in the catalytic domain. The NFT pathogenesis is less understood, however it is known that NFT derive from abnormal aggregation of hyperphosphorylated microtubule associated tau protein. However, several other pathological mechanisms are being investigated as potential contributors to AD pathology (Bonda et al., 2011; Demetrius and Driver, 2013; Femminella et al., 2013, 2014; Dineley et al., 2014). Less that 5% cases of AD are familial, with autosomical dominant mutations in *APP, PSEN1*, or *PSEN2* genes; the vast majority of AD cases are sporadic and multifactorial, andseveral genetic polymorphisms have been proposed as increased risk factors for the disease. Among these, the ε 4 allele of the Apolipoprotein E (*APOE*) is the best characterized predisposing factor for sporadic AD (Hyman et al., 2012).

In the search for reliable biomarkers in AD, great attention has focused on Aβ and tau measurement in cerebrospinal fluid (CSF) and blood, together with neuroimaging techniques looking at glucose metabolism (FDG-PET), amyloid deposition (Pittsburgh compound B-PET and derivatives) and hippocampal volume measurement on MRI (Jack and Holtzman, 2013; Femminella and Edison, 2014). However, none of these tools alone can help in diagnosis and a multi-marker approach is currently recommended. Thus, the availability of easily accessible and non-invasive biomarkers is of significant value: in this vein, microRNA (miRNA) research could represent an interesting potential.

## Basics of miRNAs

miRNAs are a novel class of small (18–25 nucleotides), single-stranded non-coding RNAs involved in the post-transcriptional regulation of gene expression. Their mechanism of action is mediated by complementar binding to the 3′ untranslated region (3′UTR) of mRNA, leading to degradation or translational repression of the target mRNA (Goodall et al., 2013). In the cellular nucleus, a primary transcript (pri-miRNA) is generated by RNA polymerase II; subsequently, the RNAseDrosha digests the pri-miRNA to release hairpin structures called pre-miRNA which are transported to the cytoplasm. Here, another RNAse called Dicer cleaves the pre-miRNA to generate double-stranded miRNA, which then binds to Argonaute (Ago) proteins. Then, only one strand is retained and associates to the RNA-induced silencing complexes (RISC) to control mRNA translation (Goodall et al., 2013).

miRNAs are involved in almost all biological processes, such as proliferation, development, apoptosis, inflammation and their expression is highly regulated, either by enzymes which stabilize mature miRNAs or by epigenetic mechanisms such as DNA methylation or histone modification. miRNAs undergo a specific tissutal and temporal distribution, as indicated by several studies on miRNA profiling in different chronic diseases and they have been found in all human biofluids. In particular, these small RNAs have demonstrated being stable in cerebrospinal fluid (CSF) and blood, probably thanks to the fact that they can be transported by liposomes or lipoproteins which prevent them from degradation (Dorval et al., 2013).

Different methods can be used to measure miRNAs levels in biofluids. Among them, the most commonly used are microarrays and quantitative PCR. While microarrays analysis is a high-throughput technique used for a non-targeted approach when several miRNAs need to be analyzed, PCR is more sensitive and usually indicated for the validation of results obtained from microarray analysis (Maciotta et al., 2013).

## miRNAs in AD

It has been demonstrated that specific miRNAs are expressed in the central nervous system (CNS), where they regulate neuronal differentiation, synaptic plasticity and neurite outgrowth. In 2007, Landgraf et al. also showed that cluster analysis based on the expression of orthologous miRNAsseparates the human adult brain regions from those of adult rat brain, with the exception of the hippocampus region; moreover, adult and embryonic brain tissues cluster separately (Landgraf et al., 2007). Several studies using profiling tecniques have shown that there is miRNAs dysregulation in AD human brain, despite some differences probably related to non-homogeneous esperimental conditions. Wang et al have specifically assessed miRNA expression in human cerebral cortical gray matter (GM) and white matter (WM) in order to provide some insights into the difference between GM and WM miRNAin AD pathology. They found that some well-characterized miRNAs were substantially enriched in WM but most of the miRNA expression variability that correlated with the presence of early AD-related pathology was seen in GM, confirming that downregulation of a set of miRNAs in GM (including several miR-15/107 genes and miR-29 paralogs) correlated strongly with the density of diffuse amyloid plaques detected in adjacent tissue. Moreover, particular subsets of miRNAswere coordinately expressed in relation to AD-related pathology, supporting the hypothesis that patterns of miRNA expression in cortical GM may contribute to AD pathogenesis (Wang et al., 2011).

In the following section we will describe some of the miRNAs that are possibly related to AD pathology and have been consistently identified as dysregulated in AD (Table 1) (Van den Hove et al., 2014).

**Table 1:** **Some of the miRNAs most commonly associated with AD**.

**MiRNA**	**Role in AD pathophysiology**	**Evidence in AD patients**	**References**
MiR-9	Addition of Aβ peptides to primary neuronal cell cultures has been shown to downregulate this small RNA. Targets include FGFR1, NFkB and SIRT1	Down regulated in patient serum	Krichevsky et al., 2003
MiR-107	Negative correlation with BACE1 and neuritic plaque density; targets BACE1, CDK5 and the metalloproteinase ADAM10	Downregulated in temporal cortex of AD patients	Nelson and Wang, 2010; Goodall et al., 2013
MiR-29	Inversely correlated with BACE1; in neuronal cellular models it has been shown to increase amyloid production *in vitro*	Downregulation in human AD temporal cortex, cerebellum and patient serum	Hebert et al., 2008
MiR-34	Regulates the expression of p53, that is associated to tau phosphorylation	High expression levels in the hippocampus of patients with AD	Hooper et al., 2007; Zovoilis et al., 2011
MiR-181	Aβ is able to downregulate MiR-181 expression in primary human astrocyes cultures; regulates SIRT1 expression	Downregulated in human temporal cortex and patient serum	Schonrock et al., 2012a,b
MiR-106	Directly bind to APP mRNA; can also regulate the expression of the transporter ABCA1, which is involved in ApoE production	Downregulated in the temporal cortex of AD patients	Kim et al., 2012
MiR-146a	Regulator of inflammation-related mRNA, acts as an inflammatory response repressor in the CNS	“Selective” upregulation in brain regions affected by AD pathology, such as temporal cortex and hippocampus	Sethi and Lukiw, 2009
MiR-155	Appears to have specific effects on complement factor H (CFH) down-regulation in neurodegenerative brain	Upregulated in Down's syndrome, a congenital human neurological disorder that shows a remarkable AD-like neuropathology with age	Lukiw and Alexandrov, 2012

miR-9: miR-9 is encoded by three different genes and is one of the most frequently altered miRNAs found in AD. It is highly expressed in fetal hippocampus while it is downregulated in AD brains; addition of Aβ peptides to primary neuronal cell cultures has been shown to downregulate this small RNA (Krichevsky et al., 2003). The targets of miR-9 include the fibroblast growth factor receptor 1 (FGFR1), NFkB and sirtuin 1 (SIRT1), a deacetylase interacting with tau and probably contributing to the formation of hyperphosphorylated forms. Moreover, miR-9 also targets the transcription factor REST, which is implicated in silencing neuronal gene expression in non-neuronal cells (Van den Hove et al., 2014). In particular, it has been demonstrated that REST contains miRNA recognition elements for miR-9 and, vice versa, miR-9 is processed from a primary transcript which has sequences that can be occupied by REST. Further evidence on the important function of miR-9 derives from studies in patients affected with Hutchinson-Gilford progeria syndrome (HGPS), who exhibit systemic premature aging without any relevant cognitive impairment. It has been shown indeed that in neuronal cells of HGPS patients, the toxic accumulation of progerin protein is prevented by the expression of miR-9 in these cells.

miR-107: this small RNA has been reported to be downregulated in temporal cortex at an early stage of AD (Nelson and Wang, 2010). Its expression has a negative correlation with BACE1 and with neuritic plaque density, as well as neurofibrillary tangles and it has been proven that it targets BACE1, thus regulating amyloid production. Moreover, it has been demonstrated that miR-107 can also modulate cyclin-dependent kinase 5 (CDK5), which is known to be dysregulated in AD, and the metalloproteinase ADAM10, involved in APP processing (Goodall et al., 2013).

miR-29: this is a family of miRNAs whose expression is inversely correlated with BACE1 and in neuronal cellular models it has been shown to increase amyloid production *in vitro* (Hebert et al., 2008). Other studies have suggested that miR-29 family might play a role in brain aging, as well as in microglial activity modulation. Among the targets of miR-29 family are the microglial modulators Insulin-like growth factor-1 (IGF-1) and fractalkine ligand (CX3CL1). Indeed, higher expression of miR-29 in the brain of aged mice was associated with reduced mRNA levels of IGF-1 and CX3CL1. Moreover, increased expression of miR-29b in human cortical tissue was negatively correlated with IGF-1 and CX3CL1 expression.

miR-34: this is also a family of miRNAs which has been implicated in several physiological processes. In AD, it seems that miR-34 regulate the expression of p53, that is associated to tau phosphorylation (Hooper et al., 2007). Moreover, miR-34 is found at high levels in the hippocampus of patients with AD and in transgenic mouse models of the disease. In these, the downregulation of miR-34 is able to rescue some cognitive abilities (Zovoilis et al., 2011).

miR-181: this miRNA has been involved in different disease pathophysiological processes as a regulator of genes such as oncogene RAS and tumor necrosis factor alpha. In AD, miR-181 is correlated with Aβ levels in human AD brain and *in vitro* experiments have shown that Aβ is able to downregulate miR-181 expression in primary humanastrocyes cultures. Moreover, miR-181 has been suggested as one of the regulators of SIRT1 expression (Schonrock et al., 2012a,b).

miR-106: Both miR-106a and miR-106b have been shown to directly bind to APP mRNA and are downregulated in the temporal cortex of AD patients. Notably, they can also regulate the expression of the transporter ABCA1, which is involved in ApoE production, suggesting that these miRNAs might have multiple roles in AD pathophysiological processes (Kim et al., 2012).

miR-146a: miR-146a is a known regulator of inflammation-related mRNA, such as the complement factor H which is downregulated in AD brains and acts as an inflammatory response repressor in the CNS. MiR-146a has also shown a “selective” upregulation in brain regions affected by AD pathology, such as temporal cortex and hippocampus, while normal levels have been found in non-affected regions (Sethi and Lukiw, 2009).

Not only those described above, but also other miRNAs have been associated with AD, such as miR-124, miR-132, and miR-153, and other neurodegenerative diseases. Most of them have shown implications in APP processing, neuroinflammation, tau phopshorilation and ApoElipidization and some of them seem to be involved in more than one of these processes, suggesting that they can also mediate cross-talk among the different pathological processes underlying AD (Goodall et al., 2013; Van den Hove et al., 2014).

## miRNAs as biomarkers in AD

To date, the diagnosis of probable AD is based on clinical and neuropsychological evaluation, structural (MRI) and functional (PET) neuroimaging techniques, and CSF determination of levels of Aβ and tau. However, this combined biomarkers approach results in a sensitivity of about 93% and specificity of 55%. Thus, several studies have tried to establish the potential role of other biomarkers and miRNAs have been extensively investigated at this aim, to be used for screening, differential diagnosis and disease progression monitoring (Jack and Holtzman, 2013). A recent systematic review has shown that 10 miRNAs display a significantly different expression in AD compared to controls in at least two studies and more than thirty miRNAs are able to distinguish between two neurodegenerative diseases (Danborg et al., 2014). The potential of miRNAs as biomarkers derives also from their unique secretory properties, as they can exert their biological effects both close by or at a distance, can regulate multiple target genes simultaneously and can affect several cell types, being delivered independently of cell-to-cell contact (Schwarzenbach et al., 2014). Indeed, extracellular miRNAs seem to derive mainly from three sources: (1) passive leakage from injured cells; (2) active secretion via microvescicles, released by almost all cell types; (3) active secretion in complexes of miRNA-associated proteins, such as Argonaute2. However, the details of these mechanisms still need further elucidation (Chen et al., 2012).

Following we would like to summarize the findings of the most significant studies that have investigated the potential role of miRNAs as biomarkers in AD (Table 1).

Schipper et al. (2007) searched for possible differences in the levels of 462 human miRNAs (from let-7 family to miR-663) isolated from blood mononuclear cells (BMC) obtained from sporadic AD patients and age-matched controls. The Authors found that plasma levels of miR-34a and 181b were upregulated in AD subjects, with miR-181b showing a higher increase in ApoE4-positive AD subjects. Interestingly, among the putative targets of the miR-34a and 181b were mRNAs encoding for Transcription/Translation and Synaptic Activity proteins; moreover, such transcripts were also targeted by miRNAs involved in the regulation of Injury response and Redox homeostasis (Schipper et al., 2007). Subsequently, Cogswell et al. performed by miRNAs profiling the expression of over 300 miRNAsin hippocampus, medial frontal gyrus, andcerebellum from early and late stage AD compared to age-matched controls, revealing that expressionchanges in key miRNAsare consistent with the regional and time-dependent features of AD pathologyand are linked through their targets to known and novel pathways of disease. These authors additionally discovered that miRNAscould be detected in CSF and that CSF miRNAs are indeed altered in AD, in particular miRNAs related to multiple disease related pathways such as immune cell differentiation and innate immunity (Cogswell et al., 2008).

Several miRNAs, including the specific pro-inflammatory miRNA-9, miRNA-125b, miRNA-146a, and miRNA-155, were found to be increased in both extracellular fluid (ECF) and CSF of AD patients. Such miRNAs derive from the cleavage of NF-κB-regulated pre-miRNA precursor, and regulate innate immune and inflammatory responses in AD brain. Interestingly, miRNA-9, miRNA-125b, miRNA-146a, miRNA-155 are also released *in vitro* by human primary neuronal-glial cells treated with ECF obtained from AD subjects. This signaling network between CSF and ECF suggest a possible role for CNS miRNAs as paracrine mediators (Alexandrov et al., 2012).

Similar findings were reported by Lukiw et al., who show that two proinflammatorymiRNAs, miRNA-146a, and miRNA-155, are abundant in ECF and in CSF of AD patients and that these miRNAs are secreted by human brain cells stressed with factors known to be elevated in AD brain. Moreover, they demonstrated that a conditioned medium containing miRNA-146a and miRNA-155, induces inflammatory gene expression in control brain cells, an effect that is mediated in part by the downregulation of the important immune system regulator CFH (Lukiw and Alexandrov, 2012). Interestingly, they further demonstrated that these effects are suppressed using anti-miRNA-146aand anti-miRNA-155, suggesting that anti-miRNA strategies may be useful in the prevention of miRNA mediated disease spreading (Lukiw et al., 2012).

Lehmann and colleagues characterized the role of the miRNA let-7b as an activator of toll-like receptor 7 (TLR7) in both immune cells and neurons. They found that CSF from individuals with AD contains increased amounts of let-7b, and extracellularintroduction of let-7b into the CSF of wild-type mice by intrathecal injection resulted in neurodegeneration. They hypothesized that miRNAs such as let-7bare released under pathological conditions and stimulate TLR7, thereby sending a “danger” signal to neurons; this may cause further spread of CNS damage in classical neurodegenerative diseases, such as AD (Lehmann et al., 2012).

Importantly, the expression levels of putative miRNAsas diagnostic markers has also been evaluated in the blood sera of AD patients, showing that miR-137, -181c, -9, -29a/b were downregulated in a subgroup of mild and severe sporadic AD as well as in mouse risk factor models (Geekiyanage et al., 2012).

## Conclusions

Circulating miRNAs are one of the most promising biomarkers for AD and for discrimination between neurodegenerative diseases. Some of them have been consistently identified as AD-specific miRNAs and their targets also seem implicated in pathophysiological processes underlying AD. However, although miRNAs are considered to be stable in biofluids, variability can be due to sample handling and processing conditions, which may induce even 1000-fold differences in specific miRNAs levels. Thus, standardization of procedures must be obtained, as well as stability profiles for each biomarker. Moreover, there is evidence that a single miRNA can regulate as many as 200 mRNAs and also one mRNA can be regulated by multiple miRNAs. This concept could be a potential limitation for a therapeutic development of miRNAs: the interference with several biological processes when using miRNA agonist/antagonist delivery could be a limitation that is difficult to overcome.

In conclusion, miRNAs have a great potential as biomarkers in diagnosis, monitoring of disease progression and therapeutic response in AD and in other neurodegenerative diseases. Further studies are needed to better understand their role in physiological and pathological conditions and to validate their use as non-invasive biomarkers.

### Conflict of interest statement

The authors declare that the research was conducted in the absence of any commercial or financial relationships that could be construed as a potential conflict of interest.
